# Accelerated rehabilitation after proximal hamstring avulsion repair is safe and effective: Outcomes from randomized controlled trial of two different rehabilitation regimes

**DOI:** 10.1002/ksa.70030

**Published:** 2025-09-09

**Authors:** Randeep S. Aujla, Peter Edwards, Steven Cecchi, Shahbaz Malik, Brendan Ricciardo, Peter Annear, Jay Ebert, Peter D'Alessandro

**Affiliations:** ^1^ University Hospitals of Leicester Leicester UK; ^2^ Curtain University School of Allied Health Perth Western Australia Australia; ^3^ Orthopaedic Research Foundation of Western Australia Perth Western Australia Australia; ^4^ Fiona Stanley Hospital Perth Western Australia Australia; ^5^ Worcestershire Acute Hospitals NHS Trust Worcester UK; ^6^ Perth Orthopaedic & Sports Medicine Centre Perth Western Australia Australia; ^7^ University of Western Australia (Exercise and Sport Science) Perth Western Australia Australia; ^8^ Medical School, Division of Surgery University of Western Australia Perth Western Australia Australia

**Keywords:** avulsion, hamstring, rehabilitation, tendon, tendon repair

## Abstract

**Purpose:**

The purpose of the study was to identify the safety and effectiveness of an accelerated (AR) versus conservative (CR) rehabilitation regimen following surgical repair of proximal hamstring tendon avulsions.

**Methods:**

This prospective randomized controlled trial (RCT) allocated patients undergoing proximal hamstring tendon repair to either a braced, partial weight‐bearing rehabilitation regime (CR = 30) or an accelerated, unbraced regime, which permitted full weight‐bearing as tolerated (AR = 27). Patients were evaluated pre‐operatively and at 6 weeks, 3 and 6 months post‐surgery, via patient‐reported outcome measures (PROMs), patient satisfaction and global rating of change (GRC) scores. Objective measures, including the single (SHD), triple (THD) and triple crossover (TCHD) hop tests, were assessed at 6 months, as was peak isokinetic knee extensor and flexor torque.

**Results:**

Of the 57 patients recruited, 4 were excluded from the CR group (2 infections; 2 re‐injuries). The CR group contained 57% males with a mean age of 45.6 (standard deviation [SD] = 13.4). The AR group contained 44% males with a mean age of 50.5 (SD = 11.8). Therefore, 53 patients (CR = 26, AR = 27) were reviewed at the final 6‐month follow‐up. Within‐group analyses showed that both groups significantly improved in all PROMs (*p* < 0.05). No significant differences were observed between groups for satisfaction, GRC scores, knee extensor torque, knee flexor torque, limb symmetry indices, re‐injuries or complications at 6 months.

**Conclusions:**

This RCT has highlighted the safety and efficacy of a post‐operative rehabilitation pathway that includes weight‐bearing as tolerated, without bracing, in patients after proximal hamstring tendon repair.

**Level of Evidence:**

Level I.

**Abbreviations**
AR: accelerated rehabilitation
BMI: body mass index
CR: conservative rehabilitation
GRC: global rating of change
LEFS: lower extremity functional status
LSIs: limb symmetry indices
MRI: magnetic resonance imaging
PHAT: Perth hamstring assessment tool
PROM: patient‐reported outcome measure
RCT: randomized controlled trial
ROM: range of motion
SHD: single hop for distance
SMD: standardized mean difference
TCHD: triple crossover hop for distance
THD: triple hop for distance
VAS: visual analogue scale
WB: weight‐bearing

## INTRODUCTION

Proximal hamstring tendon avulsions are a significant traumatic injury making up 3%–11% of all hamstring injuries [35]. Surgical treatment has been increasing since early case reports in 2005, and has now become the established primary treatment option in many institutions [10]. Multiple recent systematic reviews have demonstrated the benefit of surgical repair over non‐surgical treatment [9, 17, 35]. The largest of these meta‐analyses showed postoperative satisfaction rates of 92.6%, and good recovery of hamstring strength to 87.0% of the uninjured limb following surgery [17]. Traditional surgical indications included tendon avulsions involving all three tendons or conjoint tendon avulsions with greater than 2 cm retraction [1, 4, 13]. However, in light of growing evidence of positive surgical outcomes coupled with patients' desire for optimal function, the indications are broadening to include minimally retracted tendons and incomplete injuries [11, 21, 30].

Despite an increasing number of published studies on the topic of proximal hamstring avulsions, there is no consensus on post‐operative rehabilitation regimes [26]. A wide array of immobilization techniques, weight‐bearing restrictions and active rehabilitation regimens have been used, with many centres prescribing knee braces, hip braces or hip‐knee‐ankle orthosis in the post‐operative care of these patients [2, 5, 6, 14, 36, 40], despite their perceived limitations. A survey of protocols suggested 71% of post‐operative protocols included the use of a brace/orthosis to restrict or fix the range of motion of either the hip or knee [26]. Furthermore, the majority of protocols insist on immediate non‐weight‐bearing or toe‐touch weight‐bearing, with a mean return to full weight‐bearing of approximately 7 weeks [26]. Biomechanical studies found that the failure of suture anchor repairs of the proximal hamstring tendon occurs at 309–338 N, which is a force akin to sprinting [16]. This may suggest that the majority of current regimes are overly cautious. Accelerated rehabilitation is well established for multiple orthopaedic interventions, including lower limb arthroplasty, ankle ligament injuries, Achilles tendon ruptures, surgical stabilization of the shoulder and injuries of the knee [8, 19, 22, 31, 37]. Advantages of accelerated rehabilitation include improved blood flow to accelerate biological healing, early longitudinal strain inducing organized collagen in healing tendons/ligaments, prevention of muscle atrophy, early return of neuromuscular control and psychosocial benefits [18, 27]. These philosophies are now being applied to rehabilitation following surgical repair of proximal hamstring avulsions [3, 25].

To date, no study has been conducted to compare conservative versus more accelerated rehabilitation interventions in patients following repair of proximal hamstring tendon avulsions. We hypothesized that patients undergoing an accelerated (vs. conservative) regimen after surgical repair of acute proximal hamstring tendon avulsions, including weight‐bearing as tolerated and no bracing would: (1) demonstrate improved knee flexion strength, (2) demonstrate improved single‐limb functional hop capacity, (3) not demonstrate any differences in commonly employed patient‐reported outcome measures (PROMs) and (4) present with no detrimental effect in terms of complications and/or early re‐injury.

## MATERIALS AND METHODS

### Study design

This prospective randomized controlled trial (RCT) was registered with the Australian New Zealand Clinical Trials Register (ANZCTR: 12621000913875). Ethical approval was provided by the University of Western Australia (2021/ET000117). No patient or public involvement was involved in the study. Patients with acute proximal hamstring tendon avulsions were diagnosed upon clinical presentation to their orthopaedic surgeon (P.A., P.D. and B.R.). Patients were then assessed for their eligibility for inclusion in the study. To be included in the study, patients were between the ages of 18 and 65 years and had sustained an acute injury to their proximal hamstring tendons, involving at least two tendons, and an MRI confirming this diagnosis. Following diagnosis, patients must have been operated on within 42 days (6 weeks) of injury. Patients were excluded if they had a previous injury or surgery to the proximal hamstring tendon, had been diagnosed with an isolated semimembranosus rupture, or had a body mass index (BMI) of greater than 40 kg/m^2^. Patients were also excluded if they were unable to consent to participate or if they were unwilling to comply with their assigned rehabilitation intervention. Once patients were deemed to be eligible for participation, they were contacted and consented to by a member of the research team (J.E.). Patients were randomized in a 1:1 ratio via an online ‘random number sequence generator’. The random number sequence was maintained by a member of the research team (J.E.). A concealed allocation procedure was also used, whereby the same member of the research team had access to the randomization list. While blinding of the patient, rehabilitation therapist, and surgeon to the assigned rehabilitation protocols was not feasible, the assessor remained blinded to the allocation.

### Surgical technique

Apart from the post‐operative rehabilitation regime, all other aspects of the surgery were standardized between all lead surgeons who are fellowship trained in Orthopaedic Sports Medicine (P.A., B.R. and P.D.). Patients underwent surgical proximal hamstring tendon repair under a general anaesthetic in a prone position. Strict aseptic precautions were taken, including pre‐surgical hair removal, pre‐wash and placement of a betadine‐soaked gauze into the perineal area. Pre‐operative antibiotics were given and, following skin preparation and draping, the surgical field was sealed with an Ioban™. Transverse skin incision was used within the gluteal fold. Standard sub‐gluteal dissection down to the ischium was then performed with careful visualization, formal neurolysis and protection of the sciatic nerve throughout the procedure. Tendon mobilization was then undertaken to ensure adequate reduction under minimal tension. The lateral wall of the ischium was prepared, and repair was carried out using a minimum of three anchors in a double‐row configuration. One of the paired sutures from each first row anchor was locked into the tendon using a Krackow technique. The second suture was passed once through the tendon and used to reduce the tendon down to the lateral wall of the ischium. A second row of anchors was placed into the lateral wall to complete the double‐row repair. Following the repair, the surgical field was washed with saline lavage, and the sciatic nerve was inspected. The wounds were closed in layers following haemostasis. Skin was closed with absorbable subcuticular sutures, and a waterproof dressing was applied.

### Post‐operative rehabilitation

**Figure 1:**
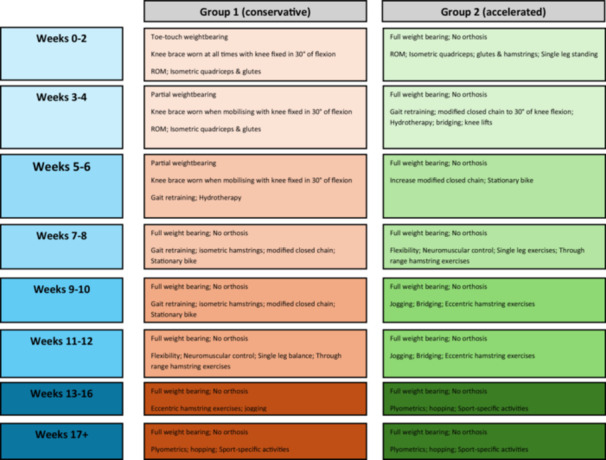
Flowchart demonstrating the two rehabilitation regimens progress at the same respective timepoints.

Patients were randomized into Group 1 (conservative rehabilitation; CR) or Group 2 (accelerated rehabilitation; AR). The rehabilitation programmes employed were developed based on a combination of existing published protocols and recommendations, and the research team's clinical experience, while further input was gathered from the university and other private therapy groups actively engaged in the treatment of patients undergoing proximal hamstring repair to ensure completeness. The relevant programme was provided to the treating therapist, with periodic communication to ensure the guide was being followed as closely as possible. While an overview of the respective rehabilitation progressions is highlighted in Table 1 (conservative group) and Table 2 (accelerated group), the primary difference between groups was the full weight‐bearing as tolerated and no use of a brace in the accelerated group, versus 6 weeks of brace use and a restricted early weight‐bearing. There was also some variation in early exercises due to the weight‐bearing (and bracing) restrictions. Direct comparison of the two regimes can be seen in Figure 1.

**Table 1:** Rehabilitation progression undertaken by the conservative rehabilitation group (Group 1), including estimated timeframes, weight‐bearing (WB) and orthosis uses, and an overview of rehabilitation activities.

Phase	Weeks	WB	Orthosis	Movement/rehabilitation overview
Protective	0–2	Toe Touch	Knee brace worn at all times (knee fixed in 30° flexion)	Initiation of passive (and active) hip and knee ROM exercises, isometric quadriceps and gluteal exercises, minimal mobilization with crutches to prevent falls.
	3–4	Partial	Knee brace worn when mobilizing (knee fixed in 30° flexion)	
	5–6			Progressive gait retraining, minimal walking with crutches only and short stride length, introducing exercises in pool.
Early	7–10	Full	Nil	Gait retraining, weight shifting exercises, single leg standing, standing knee curls and knee lifts, bridging, isometric hamstring exercises and modified closed chain exercises (including squats). Stationary bike once comfortable and hip flexion to 70° combined with knee flexion to 90° is achieved.
Progressive	11–12			Focus on flexibility and neuromuscular control, variation in single‐leg balance exercises, progression in closed‐chain exercises (including squats) to 90°, and through‐range hamstring strength exercises.
Strength	13–16			Introduction of exercises such as stationary jogging, single leg bridges, eccentric hamstring training permitted, further progression in closed chain exercises once limb control returns and pain is minimal.
Plyometric and Sport Specific	17+			Introduction of plyometrics, jump‐ and hop‐based activities as deemed relevant, and progression towards agility and other activity/sport‐specific activities.

Abbreviation: ROM, range of motion.

### Clinical outcome evaluation

The primary outcome measure was peak isokinetic knee flexor torque at 6 months post‐surgery.

Outcome measures assessing physical function included hop testing and peak isokinetic hamstring/quadriceps torque, which were performed at 6 months post‐operatively. All patients undertook a 3‐hop test battery in the following order after a warm‐up (5 min of sub‐maximal riding on a stationary bicycle followed by an optional stretching session): (1) the single hop for distance (SHD), (2) the triple hop for distance (THD), and (3) the triple crossover hop for distance (TCHD). Patients were provided verbal descriptions of each test and were permitted 2–3 warm‐up hops on each limb prior to initiating the hop battery. Each of the three hop tests was initiated on the unaffected limb and then alternated between the unaffected and operated limbs until the three valid test trials for each test were obtained. To avoid fatigue, patients were given as much time as required between trials; though this time was not standardized and was based on the individual patient's readiness to proceed. Finally, following the hop tests, the maximal isokinetic strength of the quadriceps and hamstring muscle groups was assessed via isokinetic dynamometry (Isosport International). Peak concentric knee extension and flexion strength were measured through a range of 0–90° of knee flexion, at a single isokinetic angular velocity of 90°/s and measured in nm/kg. Patients were informed that each trial would consist of four repetitions on the same leg: three low‐intensity repetitions of knee extension and flexion, immediately followed by one maximal test effort, which was recorded. Standardized verbal encouragement was provided across all trials. Each test was initiated on the unaffected leg and then alternated between the unaffected and operated limbs until three valid trials on each limb were completed. Patients were again given adequate rest in between trials to minimize fatigue.

**Table 2:** Rehabilitation progression undertaken by the accelerated rehabilitation group (Group 2), including estimated timeframes, weight‐bearing (WB) and orthosis uses, and an overview of rehabilitation activities.

Phase	Weeks	WB	Orthosis	Movement/rehabilitation overview
Protective	0–1	Full with elbow crutches	Nil	Initiation of passive (and active) hip and knee ROM exercises, isometric quadriceps and gluteal exercises, minimal mobilization with crutch as needed to prevent falls.
Early	2			Gait retraining, weight shifting exercises, single leg standing, isometric hamstring exercises and modified closed chain exercises (including squats) to 30° knee flexion.
	3			As above, with the addition of exercises such as bridging, standing knee curls and knee lifts.
	4	Full		As above, while building walking capacity and introducing exercises in the pool.
	5–6			Stationary bike once comfortable and hip flexion to 70° combined with knee flexion to 90° is achieved.
Progressive	7–8			Focus on flexibility and neuromuscular control, variation in single‐leg balance exercises, progression in closed‐chain exercises (including squats) to 90°, and through‐range hamstring strength exercises.
Strength	9–12			Introduction of exercises such as stationary jogging, single leg bridges, eccentric hamstring training permitted, further progression in closed chain exercises once limb control returns and pain is minimal.
Plyometric and Sport Specific	13+			Introduction of plyometrics, jump‐ and hop‐based activities as deemed relevant, and progression towards agility and other activity/sport‐specific activities.

Abbreviation: ROM, range of motion.

Several PROMs were assessed pre‐surgery and at 3 and 6 months post‐surgery, including: The Perth Hamstring Assessment Tool (PHAT) [7], the lower extremity functional scale (LEFS) [29], the 12‐item Short‐Form Health Survey (SF‐12), the Tegner activity scale, and a visual analogue scale (VAS) for hamstring pain frequency (VAS‐F) and severity (VAS‐S). At 6 months, a global rating of change (GRC) scale was assessed, as was patient satisfaction on a Likert response scale (1 = *very satisfied*; 2 = *somewhat satisfied*; 3 = *somewhat dissatisfied*; 4 = *very dissatisfied*). Satisfaction with the surgery overall was assessed, as was satisfaction with surgery to relieve pain, improve the ability to perform normal daily activities, and improve the ability to return to recreational activities and participate in sports. A pain diary was also used, whereby patients recorded daily pain scores and analgesia usage for the first 6 weeks.

### Sample size

A priori sample size power calculation was determined based on the recommendations of Cohen, using G‐Power. Unpublished pilot data that had been collected in patients after proximal hamstring repair within our institution had highlighted the significant side‐to‐side limb asymmetry (mean 20% asymmetry, SD 12.0) observed at 6 months post‐surgery in peak isokinetic knee flexor torque, which is consistent with recent literature that describes 6‐month knee flexor strength deficits at 6 months following surgery ranging from 17% to 26% [15]. Therefore, given our expectation that an accelerated rehabilitation regimen would restore these strength deficits at a faster rate, for an anticipated large effect size (*d* = 0.83) based on a 10% improvement in the primary outcome variable (limb symmetry index for peak concentric hamstring torque at 6 months post‐surgery), a total of 24 patients were required in each group to reveal differences at alpha 0.05 with 80% power. We aimed to recruit and clinically evaluate at least 56 patients (i.e., an additional 15% in each group) to allow for attrition over the post‐operative period.

### Data and statistical analysis

Group differences in primary outcomes were estimated using a full intention‐to‐treat analysis using a linear mixed model, utilizing data from all 27 and 30 participants allocated to the accelerated and conservative rehabilitation groups, respectively (Figure 1). The models were adjusted for baseline values of each outcome variable to ensure more accurate estimates. Missing continuous data in both groups were accounted for by restricted maximum likelihood estimation within the linear mixed models. Linear mixed models, being a likelihood‐based estimation procedure, result in non‐biased estimates when data are missing at random. This approach estimates the likely values for the missing data based on the information contained in the observed data. Group, time (as a categorical variable), group by time, and baseline values of each outcome variable were included as fixed effects. Participants were included as a random effect to account for within‐person correlation. Effect sizes for all outcomes in the primary analysis were also calculated as standardized mean difference (SMD) from estimated marginal mean and standard error estimates and interpreted according to Cohen's criteria: small (0–0.19), moderate (0.20–0.49), large (0.50–0.79) and very large (>0.8) [12]. Independent samples *t* tests were used to examine differences in normalized peak knee extensor and flexor torque values between groups at 6 months post‐surgery. Independent *t* tests were used to examine differences in knee extensor and knee flexor torque limb symmetry indices (LSIs) at 6 months post‐surgery, which were calculated by dividing the peak scores on the operated limb by those of the unaffected limb. Independent *t*‐tests were used to examine differences in SHD, THD, and TCHD at 6 months post‐surgery. Hop testing LSI's were calculated by dividing the peak scores on the operated limb by those of the unaffected limb. Mann–Whitney *U*‐tests were used to determine a difference in GRC and satisfaction scores between groups at the 6‐month follow‐up period. Analysis was performed with JASP (version 0.18.3). All tests were two‐tailed with alpha set at 0.05.

## RESULTS

**Figure 2:**
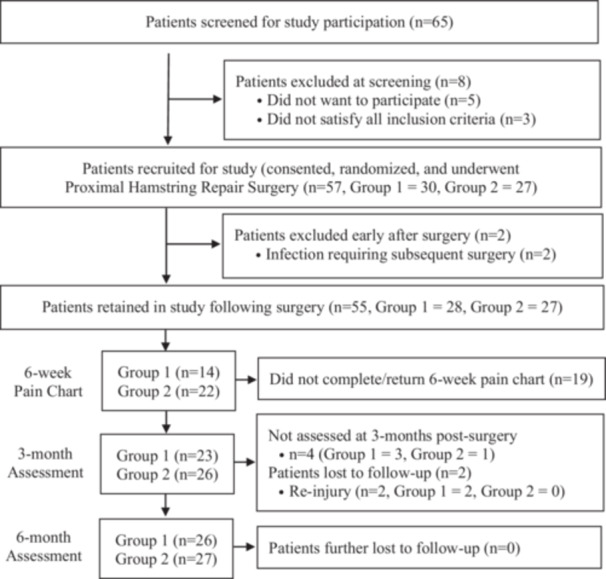
Flowchart demonstrating patient screening, recruitment, evaluation and loss to follow‐up over the study period, in patients randomized to the two post‐operative management pathways.

This study recruited 57 participants between February 2021 and March 2023 (Figure 2). Table 3 presents a comparison of baseline measures between groups for participants included in the intention‐to‐treat analysis. Groups were well matched at baseline (Table 3). Following randomization and subsequent surgery, two patients (both in the CR group) suffered post‐operative infections that also required further surgery (Figure 2). Two additional patients, also both in the CR group, had re‐injured prior to undertaking their 3‐month post‐surgery assessment (Figure 2).

**Table 3:** Baseline characteristics of the study population that were recruited, randomized and underwent surgery.

Variable	Accelerated (*n* = 27)	Conservative (*n* = 30)
Sex, males (%)	12 (44%)	17 (57%)
Age, years	50.5 (11.8), 18–65	45.6 (13.4), 21–65
Weight, kg	76.4 (14.4), 55‐102	84.9 (18.6), 49–128
BMI	27.0 (4.8), 18.7–38.5	28.0 (4.6), 17.6–38.3
Time (injury to surgery), days	23.7 (10.4), 4–42	25.6 (12.5), 4–42
Operated limb, right	16 (59%)	18 (60%)
Mechanism of injury		
Sport/recreation	13 (48%)	18 (60%)
Work/ADLs	14 (52%)	12 (40%)
PHAT	31.9 (16.2)	37.7 (17.7)
LEFS	24.9 (14.8)	33.0 (17.8)
SF‐12 (PCS)	32.4 (6.6)	34.6 (9.2)
SF‐12 (MCS)	44.8 (13.0)	49.5 (10.2)
VAS‐F	6.5 (2.4)	6.1 (3.2)
VAS‐S	6.2 (2.2)	5.1 (2.7)
Tegner	2.7 (2.1)	2.7 (2.2)

*Note*: Data are *n* (%), mean (SD) and range.

Abbreviations: ADLs, activities of daily living; BMI, body mass index; LEFI, lower extremity functional scale; PHAT, Perth hamstring assessment tool; SD, standard deviation; SF‐12 MCS, Short Form Survey Mental Component Score; SF‐12 PCS, Short Form Survey Physical Component Score; VAS‐F, visual analogue scale pain frequency; VAS‐S, visual analogue scale pain severity.

### Objective outcomes

**Figure 3:**
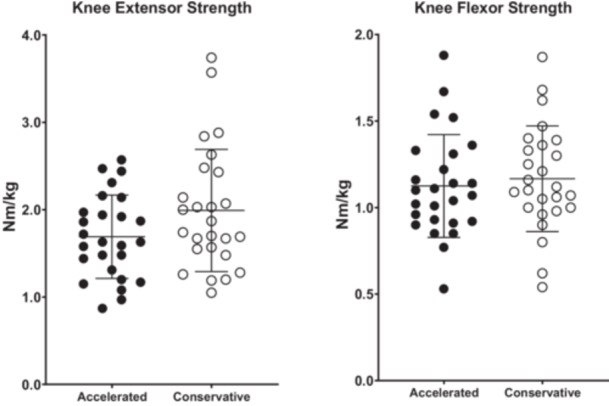
Normalized knee extensor and knee flexor torque between the accelerated and conservative rehabilitation groups, at 6 months post‐surgery. Values are mean ± SD for peak torque, normalized to body mass (nm/kg). SD, standard deviation.

**Figure 4:**
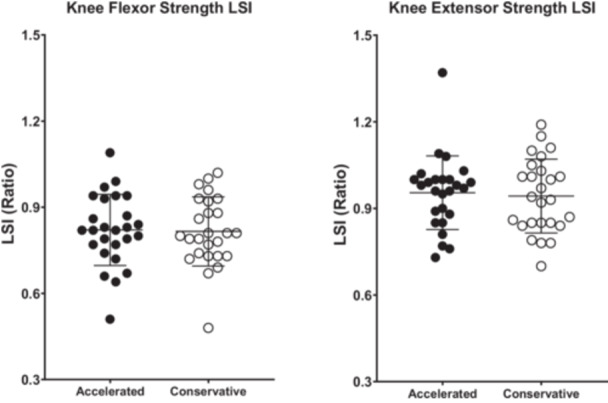
Comparisons of limb symmetry indices (LSIs) for knee flexor and knee extensor torque, calculated by the ratio of the affected side compared to the contralateral side, between the accelerated and conservative rehabilitation groups, at 6 months post‐surgery. Values are the mean ± SD for LSIs. SD, standard deviation.

No significant group differences were observed in normalized knee extensor and knee flexor torque (Figure 3), nor in LSIs of the same measures (Figure 4), at 6 months post‐surgery. No significant between‐group differences were observed in the SHD, THD, or the TCHD for both the operated and unaffected limbs at 6 months post‐surgery (*p* > 0.05). At 6 months post‐surgery, the mean LSIs in the CR group were 0.94 ± 0.08 (*n* = 22) for SHD, 0.95 ± 0.05 (*n* = 17) for THD and 0.95 ± 0.07 (*n* = 15) for TCHD, while the AR group showed mean LSIs of 0.94 ± 0.09 (*n* = 22), 0.99 ± 0.08 (*n* = 11) and 0.96 ± 0.08 (*n* = 11) for the same measures, respectively. No significant between‐group differences were observed for any LSIs in any of the hop tests at 6 months post‐surgery (*p* < 0.05).

### Subjective outcomes

**Figure 5:**
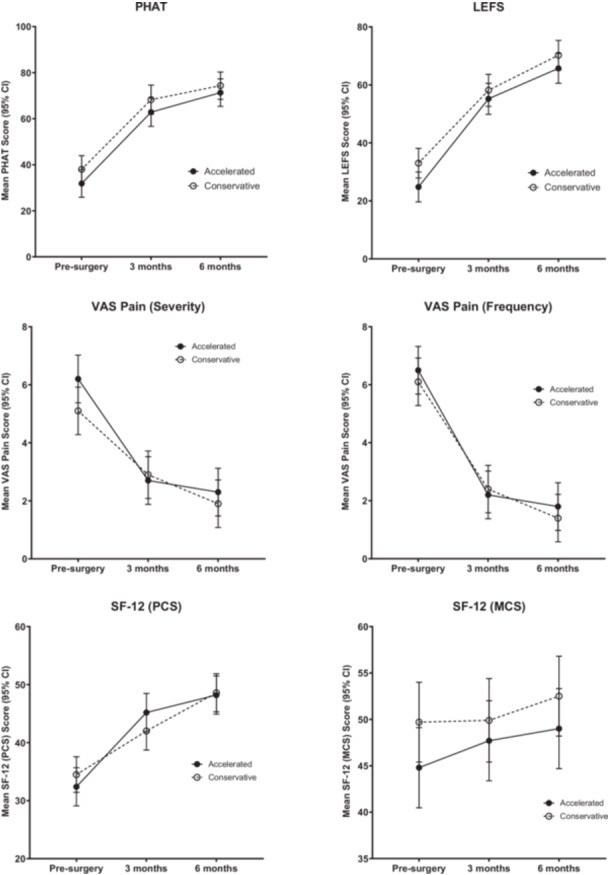
Patient‐reported outcomes for both groups pre‐surgery and at 3‐ and 6‐months post‐surgery. Higher scores represent better outcomes for all measures except for VAS pain severity and frequency. CI, confidence interval; LEFI, lower extremity functional scale; PHAT, Perth hamstring assessment tool; SF‐12 MCS, Short Form Survey Mental Component Score; SF‐12 PCS, Short Form Survey Physical Component Score; VAS, visual analogue scale.

Within‐group analyses showed that both groups significantly improved in all PROMs from baseline through to 6 months post‐surgery (*p* < 0.05) (Figure 5). There were no significant between‐group differences for any outcomes at either 3 or 6 months post‐surgery, and all SMDs were small or moderate (Figure 5, Table 4). No significant differences were observed between groups for GRC scores, or satisfaction scores, at 6 months (Table 5).

**Table 4:** Patient‐reported outcomes and rehabilitation group comparisons for estimated marginal means and between‐group differences for the intention‐to‐treat analysis.

	Accelerated	Conservative	Mean difference (95% CI)	SMD (95% CI)	*p*
PHAT					
Baseline	31.9 (2.9)	38.1 (2.9)			
3 months	62.8 (3.0)	68.5 (3.1)	−5.7 (−2.7 to 14.2)	−0.4 (−0.9 to 0.2)	0.185
6 months	71.3 (2.9)	74.8 (2.9)	−3.4 (−4.7 to 11.6)	−0.2 (−0.8 to 0.3)	0.407
LEFS					
Baseline	24.8 (2.5)	33.0 (2.5)			
3 months	55.2 (2.6)	58.2 (2.7)	−3.0 (−4.3 to 10.3)	−0.2 (−0.7 to 0.3)	0.415
6 months	65.7 (2.5)	70.0 (2.5)	−4.3 (−2.7 to 11.3)	−0.3 (−0.8 to 0.2)	0.232
SF‐12 PCS					
Baseline	32.4 (1.6)	34.5 (1.5)			
3 months	45.2 (1.6)	42.0 (1.6)	3.2 (−1.2 to 7.7)	0.4 (−0.2 to 0.9)	0.156
6 months	48.2 (1.6)	48.6 (1.6)	−0.37 (−4.7 to 3.9)	−0.04 (−0.6 to 0.5)	0.867
SF‐12 MCS					
Baseline	44.8 (2.1)	49.7 (2.1)			
3 months	47.7 (2.1)	49.9 (2.2)	−2.1 (−9.0 to 4.9)	−0.2 (−0.7 to 0.3)	0.467
6 months	49.0 (2.1)	52.5 (2.1)	−3.5 (−8.9 to 2.0)	−0.3 (−0.8 to 0.2)	0.257
VAS‐S					
Baseline	6.2 (0.4)	5.1 (0.4)			
3 months	2.7 (0.4)	2.9 (0.4)	−0.19 (−1.4 to 1.0)	−0.1 (−0.6 to 0.4)	0.754
6 months	2.3 (0.4)	1.9 (0.4)	0.39 (−0.7 to 1.5)	0.2 (0.3–0.7)	0.499
VAS‐F					
Baseline	6.5 (0.4)	6.1 (0.4)			
3 months	2.2 (0.4)	2.4 (0.4)	−0.27 (−1.4 to 0.9)	−0.1 (−0.6 to 0.5)	0.814
6 months	1.8 (0.4)	1.4 (0.4)	0.41 (−0.6 to 1.4)	0.00 (−0.5 to 0.5)	0.987
Tegner					
Baseline	2.7 (0.3)	2.7 (0.3)			
3 months	3.3 (0.3)	3.8 (0.3)	−0.41 (−1.4 to 0.5)	−0.2 (−0.8 to 0.3)	0.400
6 months	4.1 (0.3)	4.6 (0.3)	−0.48 (−1.4 to 0.5)	−0.3 (−0.8 to 0.3)	0.317

*Note*: Data are mean (SE) unless otherwise indicated.

Abbreviations: CI, confidence interval; LEFI, lower extremity functional scale; PHAT, Perth hamstring assessment tool; SD, standard deviation; SF‐12 MCS, Short Form Survey Mental Component Score; SF‐12 PCS, Short Form Survey Physical Component Score; SMD, standardized mean difference; VAS‐F, visual analogue scale pain frequency; VAS‐S, visual analogue scale pain severity.

**Table 5:** Comparison of global rating of change and satisfaction scores between the accelerated and conservative rehabilitation groups, at 6 months post‐surgery.

Outcome	Median (25th–75th percentiles)		*p*
	Accelerated	Conservative	
Global rating of change	3.0 (3.0–4.0)	3.0 (1.0–4.0)	0.361
Satisfaction (overall)	1.0 (1.0–2.0)	1.0 (1.0–2.0)	0.457
Satisfaction (pain relief)	1.0 (1.0–1.0)	1.0 (1.0–2.0)	0.556
Satisfaction (activities of daily living)	1.0 (1.0–2.0)	1.0 (1.0–2.0)	0.880
Satisfaction (recreational Activities)	2.0 (1.0–2.0)	2.0 (1.0–2.0)	0.916
Satisfaction (sports)	1.0 (1.0–1.0)	1.0 (1.0–2.0)	0.455

### Daily pain scores

**Figure 6:**
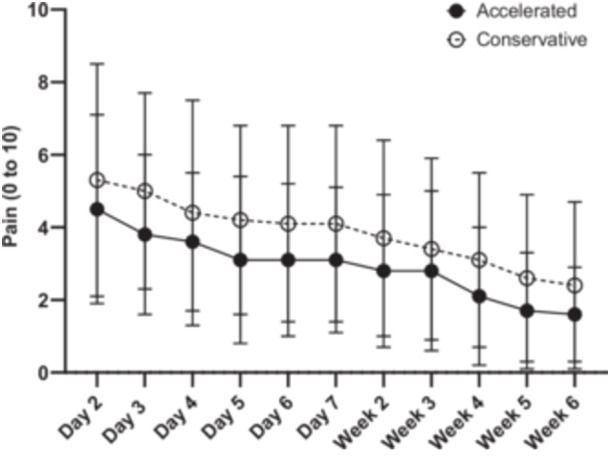
Daily self‐reported pain scores between the accelerated (*n* = 19) and conservative (*n* = 16) rehabilitation groups, over the first six post‐operative weeks. Values are mean ± SD pain scores per day. SD, standard deviation.

Figure 6 illustrates the daily self‐reported pain scores within the accelerated (*n* = 19) and conservative (*n* = 16) rehabilitation groups throughout the initial six post‐operative weeks. While both groups experienced a decline in pain intensity over time, no significant between‐group differences were observed at any measured time point during the 6‐week period.

### Complications

There were two deep infections, both occurring in patients randomized to the CR group. One case sustained an early deep infection and underwent washout and removal of implants at 3 weeks post‐operatively. The second deep infection required washout, debridement and retention of implants. There was one further early superficial wound washout (at 18 days post‐operatively) for suspected infection, which occurred in a patient randomized to the AR group. Within the 6‐month post‐operative period, two re‐ruptures occurred making an overall re‐rupture rate of 3.5%. Both re‐ruptures occurred in the CR group, including one at 6 weeks and one at 3 months, both prior to their respective 3‐month reviews, and both because of a fall/slip and re‐injury.

## DISCUSSION

The main finding of this RCT is that an accelerated rehabilitation protocol, which involves immediate full weight bearing as tolerated and no use of brace immobilization, following repair of acute proximal hamstring tendon ruptures, is safe and practical. This study presents the only RCT, to our knowledge, evaluating clinical outcomes in patients undergoing proximal hamstring tendon repairs.

However, the primary hypothesis that an accelerated rehabilitation approach would improve the peak concentric knee flexor torque at 6 months following surgery was not met. Indeed, our assessment of dynamic strength, via hop testing, also did not achieve statistical difference between the two rehabilitation regimes utilized. Despite this, the current study adds to the literature by providing data such as early strength progress via objective outcome measures.

The current study showed no difference in self‐reported pain, function and quality of life between the two groups up until 6 months following surgery. At the 3‐ and 6‐month time points, the presented study found no statistical difference in PROMs (PHAT, LEFS), VAS and Tegner scores. This finding supported our third hypothesis. However, the study was not powered to assess these outcome measures, so this may warrant further evaluation in larger studies.

Léger‐St‐Jean et al. [25] have published results on accelerated rehabilitation following proximal hamstring avulsion repair in 34 patients. Their accelerated rehabilitation involved immediate weight‐bearing and free range of motion at both the hip and knee. No brace or orthosis was used, but patients were advised against fast walking for 6 weeks. Yet despite this ‘accelerated’ rehabilitation regime, there was no formal physiotherapy initiated until 12 weeks post‐operatively. They demonstrated a mean LEFS of 87 ± 21 and a mean single assessment numeric evaluation (SANE) of 88.1 ± 11.6. Results were superior amongst the acute (<4 weeks) repairs with a mean LEFS of 93.7 ± 11.1 and a mean SANE of 91.3 ± 8.3. The acutely repaired patients were found to have a mean return to activity of 120 days. Contrasting these functional results to a wider meta‐analysis of patients, which showed an LEFS of 87.0 in 1530 patients [17]. This present study found LEFS at lower rates than this in both groups at 6 months.

Wong et al. [38] completed a retrospective review of 21 patients who underwent proximal hamstring suture anchor repair with an accelerated rehabilitation programme. They demonstrated a LEFS of 74.2 ± 7.5; SF‐12 of 51.6 ± 6.8 with no re‐ruptures at a minimum of 1‐year follow‐up. However, this ‘accelerated’ rehabilitation involved 6 weeks of touchdown weight‐bearing in a hinged knee brace locked in extension for ambulation and passive knee flexion to 90° while seated. In our opinion, their rehabilitation programme was more akin to our conservative group.

There were two re‐ruptures that occurred within our study, making an overall re‐rupture rate of 3.5%. Of interest, both re‐ruptures occurred in the CR group, although both were experienced after the early period of bracing in these patients was ceased. Nevertheless, no re‐ruptures within the first 6 post‐operative months were observed in the AR group. Given that 75% of re‐ruptures occur within the first 6 months following surgery [24], there is likely to be no increased re‐rupture rate as a result of the AR approach, relative to the CR group. This has been supported in previous studies allowing full‐weight bearing without immobilization, which documented only three re‐ruptures among 112 patients, a pooled rate of 2.7% [23, 25, 38].

Wyatt et al. [41] conducted a systematic review looking at the use of bracing following repair of proximal hamstring tendons. They compared outcomes from studies that used a bracing protocol versus those that were unbraced. They found lower complication and re‐rupture rates, as well as higher PROMs, satisfaction and return to sport rates, in the braced patients. This study investigated only one aspect of an accelerated rehabilitation protocol in terms of whether a brace was used or not. Furthermore, this systematic review is limited by the heterogeneity of the studies included. For example, the braced cohort consisted of 67% acute repairs versus 46% in the unbraced acute repairs. It is known that earlier repairs lead to improved clinical outcomes [32, 34, 39]. Also, the unbraced cohort was almost 10 years older than the braced cohort. Of the unbraced studies, two studies were not simply acute avulsions of the proximal hamstring tendons; one was conducted on adolescent bony avulsions, and one study consisted of chronic ruptures with significant posttraumatic ossification [20, 28]. One included unbraced study did not allow full weight‐bearing for 4 weeks, advocated crutches for up to 12 weeks and had no active physiotherapy for 5–7 weeks [33]. As a result, the conclusions from this study should be taken with caution regarding the influence of bracing.

### Limitations

There were some limitations within our study. Our study does represent low external validity due to its assessment and intervention of patients presenting to sports surgeons within Western Australia. Results may not be broadly applicable due to their narrow eligibility criteria for participants, demographics of the included population (e.g., age, activity level and country), tightly controlled implementation of interventions, shorter duration of follow‐up, and focus on short‐term specific outcomes. Another point to note is that the rehabilitation regimes were not overly prescriptive in their application. This was the intention of the investigators, as there was an acceptance that patients would seek post‐operative physiotherapy in their own areas, and this could not be standardised in terms of specific rehabilitation exercises given to individual patients. The rehabilitation regimes were there to provide guidelines and a homogeneous structure for progression through the post‐operative rehabilitation. Finally, this study was powered to assess peak hamstring torque at 6 months following surgical repair. It was not powered to assess for significance for PROMs or complications.

## CONCLUSION

This RCT demonstrates no difference in the primary outcome measure of peak isokinetic hamstring torque at 6 months following the institution of a conservative or accelerated rehabilitation regime after surgical repair for proximal hamstring tendon avulsions. However, no difference between rehabilitation groups was observed in PROMs, complications and/or re‐ruptures within the first 6 post‐operative months, although the study was not powered for these outcomes. We recommend that an accelerated rehabilitation regime, which involves immediate full weight bearing as tolerated and no use of brace immobilization should be considered as first‐line post‐operative management for patients following proximal hamstring repair. However, long‐term studies would add evidence about the durability of this approach.

## AUTHOR CONTRIBUTIONS

*Study design, methodological development, data collection, data analysis, writing and reviewing*: Randeep S. Aujla, Jay Ebert and Peter D'Alessandro. *Data collection, data analysis, writing and reviewing*: Peter Edwards. *Study design, data collection, data analysis and reviewing*: Steven Cecchi. *Study design, methodological development, data collection, data analysis and Reviewing*: Shahbaz Malik. *Study design, Methodological development and Reviewing*: Brendan Ricciardo and Peter Annear.

## CONFLICT OF INTEREST STATEMENT

The authors declare no conflicts of interest.

## ETHICS STATEMENT

Ethical approval was provided by the University of Western Australia (2021/ET000117).

## Data Availability

The data that support the findings of this study are available on request from the corresponding author. The data are not publicly available due to privacy or ethical restrictions.
